# Genetic and clinical characterization of Pakistani families with Bardet-Biedl syndrome extends the genetic and phenotypic spectrum

**DOI:** 10.1038/srep34764

**Published:** 2016-10-06

**Authors:** Maleeha Maria, Ideke J. C. Lamers, Miriam Schmidts, Muhammad Ajmal, Sulman Jaffar, Ehsan Ullah, Bilal Mustafa, Shakeel Ahmad, Katia Nazmutdinova, Bethan Hoskins, Erwin van Wijk, Linda Koster-Kamphuis, Muhammad Imran Khan, Phil L. Beales, Frans P. M. Cremers, Ronald Roepman, Maleeha Azam, Heleen H. Arts, Raheel Qamar

**Affiliations:** 1 Department of Biosciences, COMSATS Institute of Information Technology, Islamabad, Pakistan; 2 Department of Human Genetics, Radboud University Medical Center, Nijmegen, the Netherlands; 3 Radboud Institute for Molecular Life Sciences, Radboud University, Nijmegen, the Netherlands; 4 Genetics and Genomic Medicine, UCL Institute of Child Health, 30 Guilford Street, London, UK; 5 Center for Pediatrics and Adolescent Medicine, Pediatric Genetics Division, University Hospital Freiburg, Germany; 6 Shifa International Hospital, Islamabad, Pakistan; 7 School of Applied Sciences, Faculty of Health and Environmental Sciences, Auckland University of Technology, Auckland, New Zealand; 8 Auckland City Hospital, Auckland District Health Board, Auckland, New Zealand; 9 North East Thames Regional Genetics Service, Hospital for Children, London, UK; 10 Department of Otorhinolaryngology, Radboud University Medical Centre, Nijmegen, the Netherlands; 11 Donders Center for Neurosciences, Radboud University Nijmegen, the Netherlands; 12 Department of Pediatric Nephrology, Radboud University Medical Center, Nijmegen, the Netherlands; 13 Centre for Translational Omics-GOSgene, Genetics and Genomic Medicine, UCL Institute of Child Health, London, UK; 14 Department of Biochemistry, University of Western Ontario, London, Ontario, Canada; 15 Department of Biochemistry, Al-Nafees Medical College & Hospital, Isra University, Islamabad, Pakistan; 16 Pakistan Academy of Sciences, Constitution Avenue, Islamabad, Pakistan

## Abstract

Bardet-Biedl syndrome (BBS) is an autosomal recessive disorder that is both genetically and clinically heterogeneous. To date 19 genes have been associated with BBS, which encode proteins active at the primary cilium, an antenna-like organelle that acts as the cell’s signaling hub. In the current study, a combination of mutation screening, targeted sequencing of ciliopathy genes associated with BBS, and whole-exome sequencing was used for the genetic characterization of five families including four with classic BBS symptoms and one BBS-like syndrome. This resulted in the identification of novel mutations in BBS genes *ARL6* and *BBS5*, and recurrent mutations in *BBS9* and *CEP164*. In the case of *CEP164*, this is the first report of two siblings with a BBS-like syndrome with mutations in this gene. Mutations in this gene were previously associated with nephronophthisis 15, thus the current results expand the *CEP164*-associated phenotypic spectrum. The clinical and genetic spectrum of BBS and BBS-like phenotypes is not fully defined in Pakistan. Therefore, genetic studies are needed to gain insights into genotype-phenotype correlations, which will in turn improve the clinician’s ability to make an early and accurate diagnosis, and facilitate genetic counseling, leading to directly benefiting families with affected individuals.

Bardet-Biedl syndrome (BBS) is a complex, heterogeneous, autosomal recessively inherited disorder. The 19 causative genes that have been identified thus far^1^ encode proteins that function at different sites of the primary cilium, a non-motile oblong sensory organelle that protrudes from the surface of most mammalian cells. Within the cilium, BBS proteins are involved in a wide variety of processes ranging from regulation of intraflagellar transport to chaperonin and GTPase activity. On retinal disease information database, RetNet: Summaries of genes and loci causing retinal diseases, more than 20 genes are enlisted as causative of BBS. The BBS phenotype is multi-systemic, its primary features are blindness, renal dysfunction, intellectual disability, polydactyly, obesity and hypogonadism^2^. In addition, secondary characteristics include hepatic malfunction, type 2 diabetes mellitus, slow growth, psychomotor delay, delayed speech development, hearing loss and cardiac malformations. A BBS diagnosis can be made with the presence of at least four cardinal features, or a combination of three cardinal plus two secondary features^2^. BBS is mostly inherited as an autosomal recessive trait, but there have been a few reports that indicate oligogenic inheritance for BBS^3^,^4^,^5^, however, this mode of inheritance has been under discussion^6^,^7^,^8^,^9^.

BBS is a rare disorder with differences in the prevalence of the disease in different populations. For example, among the total live births, in North America and Europe BBS affects 1 out of 140,000–160,000^10^,^11^, but BBS is more common in Newfoundland (1/17,000)^12^ as well as in Kuwaiti bedouins (1/13,500)^13^ and the Faroe Islands (1/3,700)^14^. These differences can be due to various factors including consanguinity, which is a social norm in countries such as Kuwait, Iran, Saudi Arabia and Pakistan. At the genomic level, about 10% of the total genome has been estimated to be homozygous in such families^15^. These homozygous regions generally contain the causative genetic mutation in recessive disorders such as BBS. The genetic defect is usually inherited from a single ancestor and is passed to the father and mother of the affected child who ultimately carries identical disease-causing (homozygous) mutations in both alleles of a gene^16^,^17^,^18^. In Pakistan more than 60% of the total marriages are consanguineous, and of these, about 80% are among first cousins^16^, which explains the high frequency of homozygous mutations in families affected with recessive disorders.

The prevalence of BBS in Pakistan is not yet known. To date, there have been only nine reports of 18 Pakistani families with mutations in eight genes already known to be involved in BBS: *ARL6* (OMIM #608845)^19^,^20^, *BBS1* (OMIM #209901)^21^, *BBS2* (OMIM #606151), *BBS5* (OMIM #603650)^22^, *BBS9* (OMIM #615986)^23^, *BBS10* (OMIM #610148)^19^,^24^,^25^, *BBS12* (OMIM #610683)^22^,^26^ and *TTC8* (OMIM #608132)^27^. Therefore, comprehensive studies are needed to further explore the genetic spectrum of BBS in the Pakistani population.

In this study, four families with classical BBS and one family with a BBS-like phenotype from Pakistan were genetically analyzed. In total, four homozygous mutations were identified, including mutations in *ARL6* (F01), *BBS5* (F02 and F03), *BBS9* (F04) and *CEP164* (F05). The identified mutations in *ARL6* and *BBS5* are novel.

## Methods

### Ethics statement

In the current study the recommendations of the Helsinki declaration were followed, and the “Ethics Review Board” of the COMSATS Institute of Information Technology, Islamabad and the contributing hospitals approved the study. The recruited families were informed in detail about the purpose of the study and their written consent was taken prior to blood sample collection and genetic analyses.

### Selection and clinical evaluation of BBS families

A total of five families were included in this study, in which the diagnosis of BBS was made based on the criteria described by Beales *et al*.^2^, the phenotypes of these families are given in Table 1. Moreover, biochemical tests were performed that included urine (routine examination), serum creatinine and serum urea to evaluate renal function, gonadotropin levels were assessed to find indications of hypogonadism, and thyroid levels were analyzed to diagnose hypothyroidism (Supplementary Table S1). Abdominal and pelvic ultrasonography was also performed to assess anatomy of the vital organs, i.e. liver and kidneys. Cardiac function evaluation was done by electrocardiography (ECG). For the diagnosis of ocular abnormalities refraction testing, visual acuity testing and funduscopy were performed. Magnetic resonance imaging (MRI) for the assessment of brain anomalies were performed for one family (F05).

### DNA isolation

Blood samples of the affected and healthy individuals of BBS families were collected in ethylenediaminetetraacetic acid (EDTA) vacutainers. Genomic DNA was extracted from lymphocytes using a previously described standard protocol^28^ and then stored at −20 °C until further use.

### Targeted mutation screening

BBS probands were first screened for previously reported mutations occurring in BBS-associated genes in Pakistani patients (Supplementary Table S2)^29^. The exons in which mutations have been reported previously, were amplified and analyzed by Sanger sequencing using dye-termination chemistry (BigDye Terminator, version 3 on a 3730 or 2100 DNA analyzer; Applied Biosystems, Foster City, CA).

### Targeted exome sequencing (TES) of BBS genes

DNA of probands of whom no molecular diagnosis could be obtained after targeted mutation screening, were analyzed for mutations in 21 ciliopathy genes associated with BBS using Fluidigm Technology (Supplementary Table S3). The targeted-exome sequencing (TES) analysis was performed on a next-generation sequencing MiSeq platform as previously reported^30^,^31^.

### Whole exome sequencing and *in silico* predictions

The DNA of proband V:2 of BBS family F05 in whom no causative mutation was identified by TES was further analyzed by whole-exome sequencing (WES) using an Illumina HiSeq2000 platform. Genomic DNA from the proband was purified with a QIAamp DNA mini kit (cat# 51304) according to the manufacturer’s instructions. WES was performed at the Beijing Genomics Institute (BGI). The Agilent SureSelect version 4 exome kit was used for whole exome capture and a set of Illumina HiSeq 2 × 100 bp reads was generated. 72,192 reads were uniquely mapped to gene-coding regions and the exome had a median coverage of 50x. Variants were prioritized from WES data by using dbSNP (Feb 2009 build, GRCh37/hg19) and an in-house SNP database consisting of 5,036 exomes. Variants were selected if they occurred at <0.5% in the above-mentioned databases. The data were further prioritized by selecting truncating variants, splice site variation (until positions +6/−6) and missense variants with a Grantham score of ≥80 and/or PhyloP ≥2.7 and/or PHRED scaled Combined Annotation–Dependent Depletion (CADD-PHRED) score >15, which means that the variant is ranked among the top 5% of deleterious variations^32^. The cut-off values for the Grantham and PhyloP were based on a report of Vissers *et al*.^33^. The pathogenicity of variants was also assessed by various *in silico* programs including Polymorphism Phenotyping version 2 (Polyphen-2)^34^, Sorting Intolerant From Tolerant (SIFT)^35^ and Mutation Taster^36^. Supplementary Fig. S1 summarizes the filtering protocol. Homozygous regions in WES data were identified by using the homozygosity mapper^37^. CNV analysis was also performed on the WES data using the software Copy Number Inference From Exome Reads “CoNIFER version 0.2.2”^38^. WES reads with mapping quality (MAPQ) score >20 were prioritized and respective binary sequence alignment map (BAM) files were used for CNV detection.

### Mutation segregation analysis

Prioritized variants were first confirmed by Sanger sequencing followed by segregation analysis in the respective families.

### Splice site prediction

The *ARL6* synonymous mutation (c.534A > G; p.(Q178Q)) was identified in family F01 by TES. The effect of this synonymous change on splicing was assessed *in silico* using Alamut visual Version 2.7.1 from Interactive Biosoftware (http://www.interactive-biosoftware.com) (Supplementary Fig. S2).

### Minigene splicing assay

**Figure 1:**
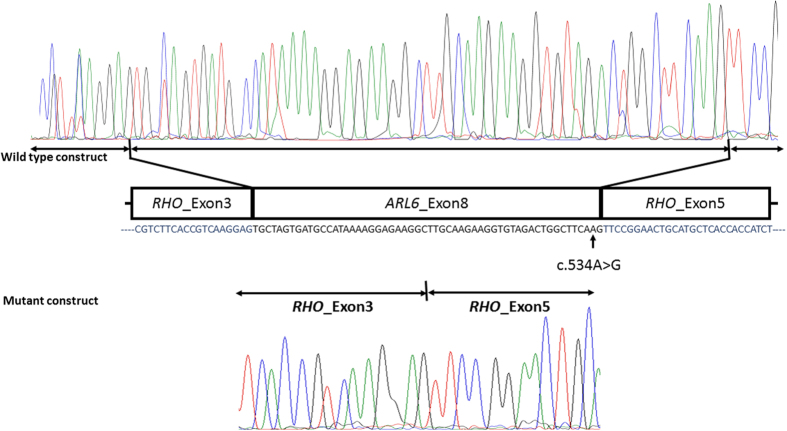
Sequencing electropherograms of the minigene splicing assay constructs in family F01. The transcriptome analysis of transfected HEK293T cells revealed that in wild-type construct *ARL6* exon 8 was retained in the transcript (upper panel) whereas in mutant construct *ARL6* exon 8 skipped splicing and was absent in the transcript (lower panel). Thus, this synonymous mutation indeed results in aberrant splicing in family F01.

The predicted effect of splice site mutation in *ARL6* (c.534A > G; p.(Q178Q)) was validated by *in vitro* experiments using a minigene assay as previously described by Cooper^39^. The amplified DNA fragments of 656 bp carrying *ARL6* exon 8 along with its flanking intronic sequences were cloned between *RHO* exon 3 and exon 5 in pCIneo mammalian expression vector. HEK293T cells in passage 20 were transfected using polyethylenimine (PEI) in Dulbecco’s modified Eagle’s medium (DMEM) supplemented with 10% fetal calf serum (FCS), 1% pyruvate and 1% antibiotic mixture of penicillin and streptomycin followed by 48 hours of incubation at 37 °C. Nucleospin kit (RNA-MACHEREY-NAGEL-05/2014, Rev 16) was used for total RNA isolation, and iSCRIPT (BioRad) RT-PCR kit was used to perform reverse transcriptase (RT) PCR. Rhodopsin exon 3 forward and exon 5 reverse primers (Supplementary Table S4) were used to detect the effect of the variant on splicing. The amplified fragments were electrophoretically separated on agarose gel followed by purification and Sanger sequencing (Fig. 1).

## Results

Families F01, F02, F03 and F04 had classical BBS phenotypes, whereas family F05 was diagnosed as having BBS-like symptoms (Table 1).

### Pre-screening of known mutations

None of the previously reported mutations in *BBS1*, *BBS2*, *BBS5*, *BBS10*, *BBS12*, *ARL6* and *TTC8* (Supplementary Table S2) in Pakistani BBS patients^29^, were found in the current panel and were therefore excluded as the causative factor in these families.

### TES revealed mutations in *ARL6*, *BBS5* and *BBS9*

**Figure 2:**
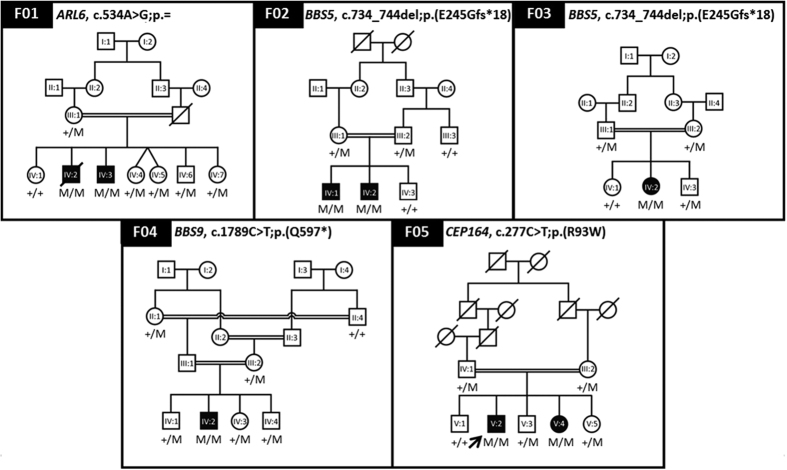
Pedigrees showing identified mutations segregating in BBS families. Squares and circles represent males and females, respectively. Unfilled symbols indicate healthy individuals and filled symbols indicate affected individuals, symbols with a diagonal line represent deceased individuals. ‘M’ represents the mutant allele and ‘+’ represents ancestral allele. Arrow in pedigree F05 indicates the individual analyzed by WES.

The TES analysis of the 21 previously reported ciliopathy genes associated with BBS (Supplementary Table S3) resulted in the identification of a novel synonymous variant c.534A > G; p.(Q178Q) in *ARL6* in family F01, and a novel 11 bp deletion c.734_744del; p.(E245Gfs*18) in *BBS5* in families F02 and F03 (Fig. 2). A recurrent mutation in *BBS9*, c.1789C > T; p.(Q597*)^40^, was identified in family F04 (Fig. 2; Table 2). All three variants segregated with the disease phenotype in the respective families, which means that the affected individuals in these families were homozygous for the detected mutations while their unaffected relatives were not. TES analysis revealed a heterozygous mutation in *BBS12*, (c.2014G > A; p.(A672T)) in family F05, which did not segregate in this family (Table 2), in addition no CNVs were detected, therefore family F05 was further analyzed by WES.

### Functional validation of the *ARL6* synonymous variant c.534A > G; p.(Q178Q)

*In silico* analysis using splice prediction tools predicted that the synonymous change c.534A > G; p.(Q178Q) found in *ARL6* in F01, could affect the normal splicing of exon 8 (Supplementary Fig. S2). The predicted effect on splicing of exon 8 was validated with a minigene splice assay, which confirmed that the c.534A > G is a variant that causes skipping of exon 8 of *ARL6* (Fig. 1). Exon skipping in turn results in a premature stop codon at residue 160 (p.(C160*)).

### WES identified a *CEP164* variant in family F05

The variants identified by WES analysis (Table 3) of family F05 were selected after *in silico* analyses, out of which the recurrent missense mutation c.277C > T; p.(R93W) in *CEP164*^41^ segregated with the disease phenotype in this family (Fig. 2; Supplementary Fig. S1; Table 3).

### LOVD mutation updates

The identified variants in the respective families were not found in the control individuals. We have uploaded all the variants in the respective Leiden Open (source) Variation Databases (LOVDs), uploaded data are available at http://databases.lovd.nl/shared/genes/ARL6, http://databases.lovd.nl/shared/genes/BBS5, http://databases.lovd.nl/shared/genes/BBS9 and http://databases.lovd.nl/shared/genes/CEP164. As an alternate LOVD gene specific data can be viewed by typing www.lovd.nl/ followed by gene symbol.

## Discussion

In the current study genetic characterization of five families from Pakistan are reported, four families had classic BBS and one family was diagnosed with a BBS-like syndrome. Two novel causative homozygous variants were identified in *ARL6* and *BBS5* in three families F01, F02 and F03, and previously reported variants were detected in *BBS9* and *CEP164* in the remaining two families F04 and F05, respectively.

In family F01, a novel synonymous variant c.534A > G; p.(Q178Q) was identified in exon 8 of *ARL6*. Although this variant does not affect the glutamine codon, *in silico* analysis predicted this mutation to result in aberrant splicing (Supplementary Fig. S2). As adenine at position 534 is the second last nucleotide in exon 8, and is located in the consensus splice site sequence, the transition of this adenine to a guanine will likely result in exon-skipping during splicing. Result of an *in vitro* splicing assay confirmed the generation of mutated mRNA and provides support for the pathogenic nature of this novel variant. Exon 8 of *ARL6* indeed appeared to be skipped during the splicing process, which results in a frameshift that causes a premature stop codon at position 160 (p.(C160*)) that may lead to the production of a truncated ARL6 peptide. However, aberrantly spliced mRNAs containing premature stop codons are usually degraded in a process that is known as nonsense mediated decay (NMD)^42^,^43^. However, when analyzing the phenotypes of the affected siblings in F01 (IV:2 and IV:3) having the common genetic defect, it is obvious that besides clinical overlap there are also clinical differences between the siblings. For example, proband (IV:3) had no renal disease, whereas his affected brother (IV:2) died because of renal failure at the age of 35 (Table 1). In addition, the degree of polydactyly varies between the siblings, i.e. while IV:2 had postaxial hexadactyly of both hands and feet, IV:3 only displayed hexadactyly of both feet.

In families F02 and F03, a novel frameshift mutation c.734_744del; p.(E245Gfs*18) was found in exon 9 of *BBS5*. The frameshift probably creates a null allele since the mRNA is likely to be degraded by NMD. In case of the synthesis of a truncated BBS5 protein, 78 amino acid residues from the C-terminus will be missing, which might affect BBSome assembly^44^,^45^ that in turn is likely to disturb normal ciliary transport mechanisms. Both families (F02 and F03) originate from the same region in Pakistan and we hypothesized that these families may be related to each other. However, both families had no information on their possible relationship. Despite having the same mutation, phenotypic variability was apparent within and between both families. Within family F02, proband (IV:1) has post axial hexadactyly on left foot, syndactyly of middle and ring finger on left hand and brachydactyly of both feet, whereas his affected sibling (IV:2) did not (Table 1). With respect to the clinical variation between families, it is apparent that the affected female in F03 did not have intellectual disability or renal anomalies, while these features were reported in both affected individuals of family F02 (Table 1). In addition, Table 1 shows various other phenotypic differences between families and patients. As observed for F01, phenotypic variability within and between F02 and F03 may be attributed to yet unknown modifying factors^21^,^46^. Generally, the existence of modifier alleles imposes a great challenge in establishing clear-cut genotype-phenotype correlations and complicates an accurate prognosis for affected individuals.

In family F04 a previously reported nonsense mutation c.1789C > T; p.(Q597*) was identified in *BBS9* by TES. This mutation had previously been reported in a homozygous state as the primary cause of a form of hereditary blindness known as retinitis pigmentosa (RP) in a Latino proband^40^. Other criteria for BBS in the Latino proband were not determined while the current proband IV:2 from family F04 was fully characterized and diagnosed with BBS (Table 1). With incomplete data of the Latino proband, the phenotypes cannot be compared between the current and the previous study. At the molecular level, as a result of this mutation, *BBS9* transcripts in this family (F04) are likely to be degraded by NMD. Alternatively, synthesis of a truncated protein may lead to disrupted BBSome assembly^47^, ciliary biogenesis and function^48^.

Based on results from previous studies and our current study, eight out of the 21 BBS-associated genes have been found mutated in Pakistani patients with BBS features (Supplementary Fig. S3). Thus far, 44 patients from 22 BBS-families^19^,^20^,^21^,^22^,^23^,^24^,^25^,^26^,^27^,^49^, including four BBS families from the current study, have been identified in Pakistan. In this group, *BBS10* mutations were most common and were identified in 27% of the cases, and mutations in *BBS5* were the second most common and explained 18% of the reported BBS families (Supplementary Fig. S3).

This analysis does not include the BBS-like family F05 from the present study. Affected individuals in family F05 were initially suspected of BBS based on the co-occurrence of RP, obesity, intellectual disability and hypogonadism in this family (Table 1). As F05 is a consanguineous family, homozygosity mapping was performed on the WES data, which revealed the presence of candidate gene *CEP164* in the fourth-largest homozygous region of 16 Mb (Supplementary Table S5). This was an interesting observation as this gene had previously been associated with Nephronophthisis (NPHP) 15 [OMIM: #614845] that is characterized by various ciliopathy features^41^. WES analysis of proband V:2 indeed identified a homozygous missense mutation c.277C > T; p.(R93W) in *CEP164* in family F05. Although structural information of the CEP164 protein remains absent to date, the affected amino acid is just downstream of the predicted WW domain of the protein (aa 57-89) that is known to mediate protein-protein interactions and might affect its structure^50^. The p.(R93W) variant had previously been reported in a compound heterozygous state together with a truncating mutation c.1573C > T; p.(Q525*) on the second allele in a family with three affected individuals of whom two presented with NPHP and blindness, while the third patient also had mild intellectual disability^41^. The F05 probands also had neural defects; V:2 had mild cerebral atrophy. In addition, psychological problem was observed in V:4 who developed obsessive compulsive disorder and psychosis at the age of 26 years. Remarkably, while all but one family with *CEP164* defects (c.277C > T; p.(R93W) and other mutations) reported by Chaki *et al*.^41^ had renal insufficiency during childhood; the affected individuals in F05 did not have any sign of renal anomalies at 21 and 26 years of age (Table 1), respectively. A single patient without renal insufficiency reported by Chaki *et al*.^41^ was described as a nonsyndromic RD (LCA) patient, a mild phenotype compared with the syndromic features of other patients carrying mutations in *CEP164* and likely caused by the specific missense mutation of the *CEP164* stop codon in this patient. Family F05 in our study was thus diagnosed with BBS-like syndrome instead of NPHP15 or BBS. It is interesting to note that Chaki *et al*.^41^, also reported a patient with a homozygous p.(R576*) mutation who showed a broader syndromic phenotype that included cerebellar vermis hypoplasia, bilateral polydactyly, abnormal liver function and obesity. In conclusion, our results confirm that mutations in *CEP164* can result in a broadly variable clinical outcome between and within families, varying from non-syndromic retinal degeneration to a BBS-like phenotype, which implies that there are restrictions on making an accurate diagnosis and prognosis in these families. Molecularly, the phenotypic resemblance with BBS is in line with the shared direct molecular association of both CEP164 and the BBSome with Rabin8, which mediates membrane assembly of the primary cilia^50^,^51^.

## Conclusion

Our data confirm inter-/intra-familial clinical heterogeneity in patients having common genetic defects in BBS genes, and describe a broader clinical phenotypic spectrum resulting from mutations in *CEP164*, extending it beyond retinal-renal ciliopathies to a BBS-like phenotype. Further studies are needed to establish if certain phenotypic features are associated with dysfunction of specific BBS genes, and if phenotypic differences between and within families can be explained by genetic modifiers. These molecular insights are helpful in genetic counseling of the affected families to prevent disease inheritance in the next generations.

## Additional Information

**How to cite this article**: Maria, M. *et al*. Genetic and clinical characterization of Pakistani families with Bardet-Biedl syndrome extends the genetic and phenotypic spectrum. *Sci. Rep.* **6**, 34764; doi: 10.1038/srep34764 (2016).

## Supplementary Material

Supplementary Information

## Figures and Tables

**Table 1:** Clinical features of affected individuals from BBS families.

Family ID	Pedigree ID	Diagnosis	Gender	Age (yrs) at diagnosis	BMI	CRD/RP	Polydactyly	Obesity	Intellectual disability	Hypogonadism	Renal anomalies	Additional features
F01	IV:2	BBS	M	32	ND	Yes	Yes	ND	No	Yes	Renal parenchymal disease; deceased due to renal failure	NA
	IV:3		M	40	ND	Yes	Yes	ND	No	Yes	No	Elevated liver enzymes, abnormal ECG, gynaecomastia
F02	IV:I	BBS	M	47	25.2	Yes	Yes	No	Yes	Yes	Bilateral renal calculi	Hypodontia, syndactyly, brachydactyly, ataxia, speech disability, gall bladder calculi, mild spleno- and hepatomegaly, elevated liver enzymes, abnormally high cholesterol level
	IV:2		M	45	25.1	Yes	No	No	Yes	Yes	Bilateral renal calculi	Speech disability
F03	IV:2	BBS	F	15	26.6	Yes	Yes	No	No	Yes	No	Developmental delay, irregular menstruation, low progesterone levels, diabetes, borderline hepatomegaly with fatty infiltration, abnormally high cholesterol level, elevated liver enzymes
F04	IV:2	BBS	M	15	33.3	Yes	Yes	Yes	Yes	Yes	Left kidney: focal caliectasis in upper and interpolar region	Elevated liver enzymes, hypodontia, speech disability, gynaecomastia
F05	V:2	BBS-like	M	20	33.8	Yes	No	Yes	Yes	Yes	No	Gynaecomastia, cerebral atrophy
	V:4		F	25	32.9	Yes	No	Yes	ND	Yes	No	Irregular menstruation, Severe depression and psychosis at 26 yrs

Abbreviations: BBS: Bardet Biedl syndrome, BMI: Body mass index, CRD: Cone-rod dystrophy, ECG: Electrocardiogram, ID: Identity, NA: Not applicable, ND: Not determined, RP: Retinitis pigmentosa, Yrs: Years.

^ƨ^Also refer to Table S1.

**Table 2:** Genetic variations identified by targeted exome sequencing of 21 BBS-associated genes in Pakistani BBS families.

Family Id	Gene	Allele 1	Allele 2	Protein variant	ExAC allele frequency (Total)	SIFTƨ(score)	Polyphen V2ƨ	Tasterƨ(p-value)
F01	*ARL6*	c.534A > G	c.534A > G	p = p.(Q178Q)	1/121,316	NA	NA	NA
F02	*BBS5*	c.734_744del	c.734_744del	p.(E245Gfs*18)	0	NA	NA	NA
F03	*BBS5*	c.734_744del	c.734_744del	p.(E245Gfs*18)	0	NA	NA	NA
F04	*BBS9*	c.1789C > T	c.1789C > T	p.(Q597*)	0	NA	NA	NA
F05	*BBS12*	c.2014G > A	+	p.(A672T)	149/119,520	Del (0.0)	DC (1.0)	PrD (1.000)

Abbreviations: DC: Disease causing, Del: deleterious, PrD: Probably damaging, ExAC: Exome aggregation consortium, SIFT: Sorting intolerant from tolerant, NA: Not applicable, Polyphen V2: Polymorphism phenotyping version2.

^ƨ^In case of protein truncating mutations these values are not applicable.

**Table 3:** Filtered variants after WES analysis in family F05.

Chr	Gene	Variation	Zygosity	Depth	ExAc frequency Total	phyloP	CADD_PHRED	Grantham Score	SIFT (score)	Polyphen V2	Mutation taster (p-value)
11	*CEP164*	c.277C > T; p.(R93W)	Hom	34	1/121,408	4.165	29	101	Del (0.0)	PrD (1.000)	DC (1.0)
4	*BBS12*	c.2014G > A; p.(A672T)	Het	115	149/119,520	5.869	34	58	Del (0.0)	PrD (1.000)	DC (1.0)

Abbreviations: CADD: Combined Annotation Dependent Depletion, Chr: Chromosome, DC: Disease causing, Del: Deleterious, ExAC: Exome aggregation consortium, Het: Heterozygous, Hom: Homozygous, PhyloP: Phylogenetic p-value, Polyphen V2: Polymorphism phenotyping version2, PrD: Probably damaging, SIFT: Sorting intolerant from tolerant.
