# Supplementary material for: Genetic and clinical characterization of Pakistani families with Bardet-Biedl syndrome extends the genetic and phenotypic spectrum
Source: Sci Rep. 2016 Oct 6;6:34764. doi: 10.1038/srep34764 (PMC5052523; doi:10.1038/srep34764)
Supplement: Supplementary Information [file srep34764-s1.pdf]

Genetic and clinical characterization of Pakistani families with Bardet-Biedl syndrome extends the genetic and phenotypic spectrum

Maleeha Maria[1,2,#], Ideke J.C. Lamers[2,3,#], Miriam Schmidts[2,3,4,5], Muhammad Ajmal[1], Sulman Jaffar[6], Ehsan Ullah[7,8], Bilal Mustafa[1], Shakeel Ahmad[1], Katia Nazmutdinova[4], Bethan Hoskins[9], Erwin van Wijk[10,11], Linda Koster-Kamphuis[12], Muhammad Imran Khan[2], Phil L. Beales[4,13], Frans P.M. Cremers[2,11], Ronald Roepman[2,3], Maleeha Azam[1,*], Heleen H. Arts[2,3,14,*], Raheel Qamar[1,15,16,*]

Supplementary Table S1: The results of biochemical tests with abnormal values.

Family ID	Individual ID	Test	Result (Normal)
F01	IV:2	**RFT**	
		Urea	198 mg/dL (13-43 mg/dL)
		Creatinine	21.26 mg/dL (0.7-1.3 mg/dL)
	IV:3	**LFT**	
		ALT	45 U/L (<40 U/L)
		ALP	321 U/L (98-279 U/L)
		**LPT**	
		serum triglycerides	167 mg/dL (<160 mg/dL)
		HDL cholesterol	28 mg/dL (35-40 mg/dL)
F02	IV:1	**RFT**	
		Creatinine	0.7 mg/dL (0.7-1.3 mg/dL)
		**LFT**	
		ALT	86 U/l (<40 U/L)
		ALP	283 U/L (98-279 U/L)
		AST	63 U/L (<38 U/L)
		**LPT**	
		Serum triglycerides	350 mg/dL (<160 mg/dL)
		Serum cholesterol	204 mg/dL (<200 mg/dL)
		HDL cholesterol	30 mg/dL (35-40 mg/dL)
		**GTPT**	
		LH	0.58 mIU/mL (1.14-8.75 mIU/mL)
		Testosterone	0.39 ng/mL (1.66-8.77 ng/mL)
		**Lactate**	
		Plasma Lactic acid	22.5 mg/dL (4.5-19.8 mg/dL)
	IV:2	**GTPT**	
		Testosterone	1.27 ng/mL (1.66-8.77 ng/mL)
F03	IV:2	**RFT**	
		Creatinine	0.7 mg/dL (0.7-1.3 mg/dL)
		**LFT**	
		ALT	50 U/l (<40 U/L)
		ALP	368 U/L (98-279 U/L)
		**LPT**	
		Serum triglycerides	253 mg/dL (<160 mg/dL)
		Serum cholesterol	243 mg/dL (<200 mg/dL)
		LDL cholesterol	155 mg/dL (100-130 mg/dL)
		**GTPT**	
		Progesterone	0.1 ng/mL (1.2-15.9 ng/mL)

F04	IV:2	**LFT**	
		ALT	40 U/l (<40 U/L)
		ALP	462 U/L (98-279 U/L)
		**LPT**	
		LDL cholesterol	90 mg/dL (100-130 mg/dL)
		HDL cholesterol	27 mg/dL (100-130 mg/dL)
		**GTPT**	
		Prolactin	32.63 ng/mL (3.46-19.40 ng/mL)
		**Lactate**	
		LDH	329 U/L (125-243 U/L)
		Plasma Lactic acid	20.1 mg/dL (4.5-19.8 mg/dL)
F05	V:2	**RFT**	
		Creatinine	1.3 mg/dL (0.7-1.3 mg/dL)
		**LFT**	
		ALT	55 U/l (<40 U/L)
		ALP	340 U/L (98-279 U/L)
		**LPT**	
		Serum cholesterol	90 mg/dL (100-130 mg/dL)
		Serum triglycerides	180 mg/dL (100-130 mg/dL)
		HDL cholesterol	34 mg/dL (100-130 mg/dL)
		**GTPT**	
		Testosterone	0.0029 ng/mL (1.66-8.77 ng/mL)
	V:4	**GTPT**	
		Testosterone	0.0008 ng/mL (1.66-8.77 ng/mL)

Abbreviations: ALP: Alkaline Phosphatase, ALT: Alanine Transaminase, AST: Aspartate aminotransferase, GTPT: Gonadotropin profile Tests, HDL: High density lipoprotein, ID: Identity, LDH: Lactate Dehydrogenase, LDL: Low Density Lipoprotein, LFT: Liver Function Tests, LH: Luteinizing Hormone, LPT: Lipid Profile Tests, RFT: Renal Function Tests

Supplementary Table S2: Targeted mutations prescreened in five probands from affected families. These mutations were reported from Pakistani BBS population (Khan et al.[29]).

Gene	RefSeq Id	Nucleotide variant	Protein variant	Exons	References
BBS1	NM_02464.9.4	c.47+1G>T	p.(?)	Ex1	1
BBS1	NM_02464.9.4	c.442G>A	p.(D148N)	Ex5	2
BBS10	NM_024685.3	c.271dup	p.(C91Lfs*5)	Ex2	3
BBS10	NM_024685.3	c.1091del	p.(N364Tfs*5)	Ex2	3
BBS10	NM_024685.3	c.1958_1967del	p.S653Ifs*4	Ex2	4
BBS10	NM_024685.3	c.2121dup	p.(K708*)	Ex2	3
BBS12	NM_152618.2	c.1589T>C	p.(L530P)	Ex3	5
BBS12	NM_152618.2	c.2102C>A	p.(S701*)	Ex3	6
BBS2	NM_031885.3	c.1237C>T	p.(R413*)	Ex11	5
ARL6	NM_032146.3	c.123+1119del	p.(?)	Intron 4	7
BBS5	NM_152384.2	c.2T>A	p.(M1K)	Ex1	5
TTC8	NM_144596.2	c.1049+2_1049+4del	p.(?)	Ex11	8

Abbreviations: RefSeq Id: Reference sequence identity

References

1. Ajmal, M. *et al.* Exome sequencing identifies a novel and a recurrent BBS1 mutation in Pakistani families with Bardet-Biedl syndrome. *Mol Vis* **19**, 644-53 (2013).
2. Beales, P.L. *et al.* Genetic interaction of BBS1 mutations with alleles at other BBS loci can result in non-Mendelian Bardet-Biedl syndrome. *Am J Hum Genet* **72**, 1187-99 (2003).
3. White, D.R. *et al.* Autozygosity mapping of Bardet-Biedl syndrome to 12q21.2 and confirmation of FLJ23560 as BBS10. *Eur J Hum Genet* **15**, 173-8 (2007).
4. Agha, Z. *et al.* A novel homozygous 10 nucleotide deletion in BBS10 causes Bardet-Biedl syndrome in a Pakistani family. *Gene* **519**, 177-81 (2013).
5. Harville, H.M. *et al.* Identification of 11 novel mutations in eight BBS genes by high-resolution homozygosity mapping. *J Med Genet* **47**, 262-7 (2010).
6. Pawlik, B. *et al.* A Novel Familial BBS12 Mutation Associated with a Mild Phenotype: Implications for Clinical and Molecular Diagnostic Strategies. *Mol Syndromol* **1**, 27-34 (2010).
7. Chen, J. *et al.* Molecular analysis of Bardet-Biedl syndrome families: report of 21 novel mutations in 10 genes. *Invest Ophthalmol Vis Sci* **52**, 5317-24 (2011).
8. Ansley, S.J. *et al.* Basal body dysfunction is a likely cause of pleiotropic Bardet-Biedl syndrome. *Nature* **425**, 628-33 (2003).

Supplementary Table S3: Transcripts and exons of 21 BBS genes sequenced in targeted next generation sequencing panel.

Chr#: Position	Gene	Transcript	Exons
chr3:97486937-97516899	*ARL6*	NM_032146	2, 3, 4, 5, 6, 7, 8
chr3:97486937-97516899		NM_177976	2, 3, 4, 5, 6, 7, 8
chr10:112660235-112677929	*BBIP1*	NM_001195304	0, 1, 2, 3
chr10:112660111-112677929		NM_001195305	0, 1, 2
chr10:112660111-112677929		NM_001195306	0, 1, 2
chr10:112660111-112677929		NM_001195307	0, 1
chr12:76739586-76742152	*BBS10*	NM_024685	0, 1
chr4:123663033-123665186	*BBS12*	NM_001178007	2
chr4:123663033-123665186		NM_152618	1
chr11:66278116-66278183	*BBS1*	NM_024649	0, 1, 2, 3, 4, 5, 6, 7, 8, 9, 10, 11, 12, 13, 14, 15, 16
chr16:56518666-56553788	*BBS2*	NM_031885	0, 1, 2, 3, 4, 5, 6, 7, 8, 9, 10, 11, 12, 13, 14, 15, 16
chr15:73016911-73029934	*BBS4*	NM_001252678	6, 7, 8, 9, 10, 11, 12, 13, 14
chr15:72978554-73029934		NM_033028	0, 1, 2, 3, 4, 5, 6, 7, 8, 9, 10, 11, 12, 13, 14, 15
chr2:170336049-170361098	*BBS5*	NM_152384	0, 1, 2, 3, 4, 5, 6, 7, 8, 9, 10, 11
chr4:122749289-122791482	*BBS7*	NM_018190	0, 1, 2, 3, 4, 5, 6, 7, 8, 9, 10, 11, 12, 13, 14, 15, 16, 17

chr4:122747008-122791482		NM_176824	0, 1, 2, 3, 4, 5, 6, 7, 8, 9, 10, 11, 12, 13, 14, 15, 16, 17, 18
chr7:33185850-33644844	*BBS9*	NM_001033604	1, 2, 3, 4, 5, 6, 7, 8, 9, 10, 11, 12, 13, 14, 15, 16, 17, 19, 20, 21
chr7:33185850-33644844		NM_001033605	1, 2, 3, 4, 5, 6, 7, 8, 9, 10, 11, 12, 13, 14, 15, 16, 17, 19, 20, 21
chr7:33185850-33644844		NM_014451	1, 2, 3, 4, 5, 6, 7, 8, 9, 10, 11, 12, 13, 14, 15, 16, 17, 19, 20
chr7:33185850-33644844		NM_198428	1, 2, 3, 4, 5, 6, 7, 8, 9, 10, 11, 12, 13, 14, 15, 16, 17, 19, 20, 21, 22
chr1:32667522-32670855	*CCDC28B*	NM_024296	1, 2, 3, 4, 5
chr12:88442954-88535098	*CEP290*	NM_025114	0, 1, 2, 3, 4, 5, 6, 7, 8, 9, 10, 11, 12, 13, 14, 15, 16, 17, 19, 20, 21, 22, 23, 24, 25, 26, 27, 28, 29, 30, 31, 32, 33, 34, 35, 36, 37, 38, 39, 40, 41, 42, 43, 44, 45, 46, 47, 48, 49, 50, 51, 52
chr22:37154348-37171765	*IFT27*	NM_001177701	0, 1, 2, 3, 4, 5, 6
chr22:37154348-37171765	*IFT27*	NM_006860	0, 1, 2, 3, 4, 5, 6
chr3:45867799-45883497	*LZTFL1*	NM_020347	0, 1, 2, 3, 4, 5, 6, 7, 8, 9
chr20:10385888-10394176	*MKKS*	NM_018848	0, 1, 2, 3
chr20:10385888-10394176		NM_170784	0, 1, 2, 3
chr17:56283433-56296886	*MKS1*	NM_001165927	0, 1, 2, 3, 4, 5, 6, 7, 8, 9, 10, 11, 12, 13, 14, 15, 16, 17
chr17:56283433-56296605		NM_017777	0, 1, 2, 3, 4, 5, 6, 7, 8, 9, 10, 11, 12, 13, 14, 15, 16, 17
chr2:63349134-63665031	*WDPCP*	NM_001042692	0, 1, 2, 3, 4, 5, 6, 7, 8, 9, 10, 11
chr3:45867799-45879509	*LZTFL1*	NM_001276378	0, 1, 2, 3, 4, 5, 6, 7, 8

chr3:45867812-45954652		NM_001276379	0, 1, 2, 3, 4, 5, 6, 7, 8
chr3:97486937-97516899	*ARL6*	NM_001278293	1, 2, 3, 4, 5, 6, 7
chr14:89291037-89343760	*TTC8*	NM_001288781	1, 2, 3, 4, 5, 6, 7, 8, 9, 10, 11, 12, 13, 14, 15
chr14:89307857-89343760		NM_001288782	5, 6, 7, 8, 9, 10, 11, 12, 13
chr14:89323526-89343760		NM_001288783	8, 9, 10, 11, 12, 13, 14
chr1:32667522-32670405	*CCDC28B*	NM_001301011	1, 2, 3, 4
chr1:243419461-243663093	*SDCCAG8*	NM_006642	0, 1, 2, 3, 4, 5, 6, 7, 8, 9, 10, 11, 12, 13, 14, 15, 16, 17
chr8:94770761-94828686	*TMEM67*	NM_001142301	2, 3, 4, 5, 6, 7, 8, 9, 10, 11, 12, 13, 14, 15, 16, 17, 19, 20, 21, 22, 23, 24, 25, 26, 27, 28
chr8:94767128-94828686		NM_153704	0, 1, 2, 3, 4, 5, 6, 7, 8, 9, 10, 11, 12, 13, 14, 15, 16, 17, 19, 20, 21, 22, 23, 24, 25, 26, 27
chr9:119460007-119461989	*TRIM32*	NM_001099679	1
chr9:119460007-119461989	*TRIM32*	NM_012210	1
chr14:89291037-89343760	*TTC8*	NM_144596	1, 2, 3, 4, 5, 6, 7, 8, 9, 10, 11, 12, 13, 14, 15
chr14:89291037-89343760		NM_198309	1, 2, 3, 4, 5, 6, 7, 8, 9, 10, 11, 12, 13, 14
chr14:89291037-89343760		NM_198310	1, 2, 3, 4, 5, 6, 7, 8, 9, 10, 11, 12, 13
chr2:63349134-63815419	*WDPCP*	NM_015910	0, 1, 2, 3, 4, 5, 6, 7, 8, 9, 10, 11, 12, 13, 14, 15, 16, 17

Abbreviation: Chr #: Chromosome number

Supplementary Table S4: Primers used for the Sanger sequencing of RT-PCR products in minigene splicing assay.

Primer	Primer Sequence 5'-3'
pCI-Neo-Rho-Insert Fw	cggaggtcaacaacgagtct
pCI-Neo-Rho-Insert Rev	aggtgtaggggatgggagac

Supplementary Table S5: Homozygosity mapping on WES data of the proband from family F05 using GRCh37/hg19 reference assembly. The cutoff selected for significant homozygous regions is 1Mb.

**Rank**	**Chr**	**From Mb-to Mb**	**Size Mb**	**Retnet genes**	**Genes from WES**
1	8	38,027,483-81,892,766	43.9	*ADAM9, RP1, TTP1, PXMP3*	
2	14	34,247,631-59,730,423	25.5		
3	12	12,243,828-29,936,501	17.7	*PDE6H*	
4	11	104,871,322-120,823,608	16.0	*C1QTNF5, MFRP*	*CEP164*
5	21	35,276,440-48,063,476	12.8		
6	6	112,671,813-124,759,146	12.1		
7	1	154,728,279-164,853,647	10.1	*SEMA4A*	
8	13	96,705,739-106,124,923	9.4		
9	4	62,861,964-71,232,388	8.4		
10	11	28,232,879-34,668,143	6.4		
11	14	89,656,709-94,908,876	5.3	*FBLN5*	
12	1	177,250,670-181,479,906	4.2		
13	17	41,466,121-45,234,407	3.8		
14	13	111,368,316-114,838,992	3.5	*GRK1*	
15	17	1,490,409-4,859,123	3.4	*PRPF8*	
16	11	46,907,827-48,346,681	1.4		
17	19	43,877,081-45,117,163	1.2		
18	11	1,630,342-2,790,019	1.2		

Abbreviations: Chr: Chromosome, Mb: Megabases, WES: Whole exome sequencing

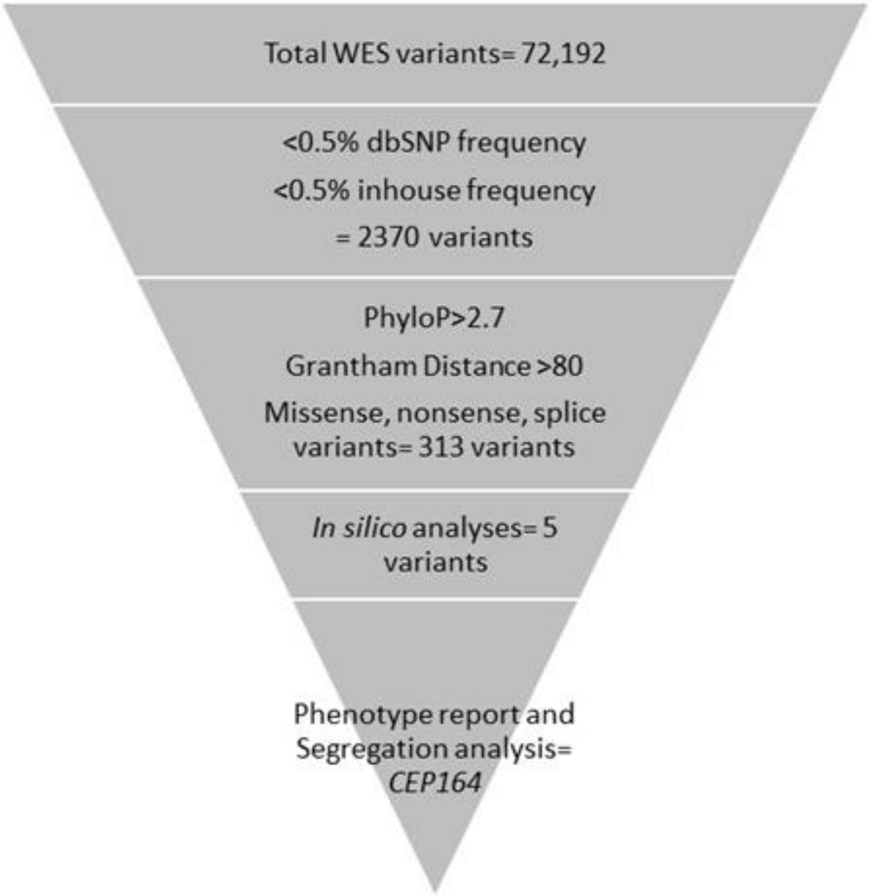

Supplementary Fig. S1: WES data filter summary for family F05.

***ARL6*, c.534A>G; p.=**

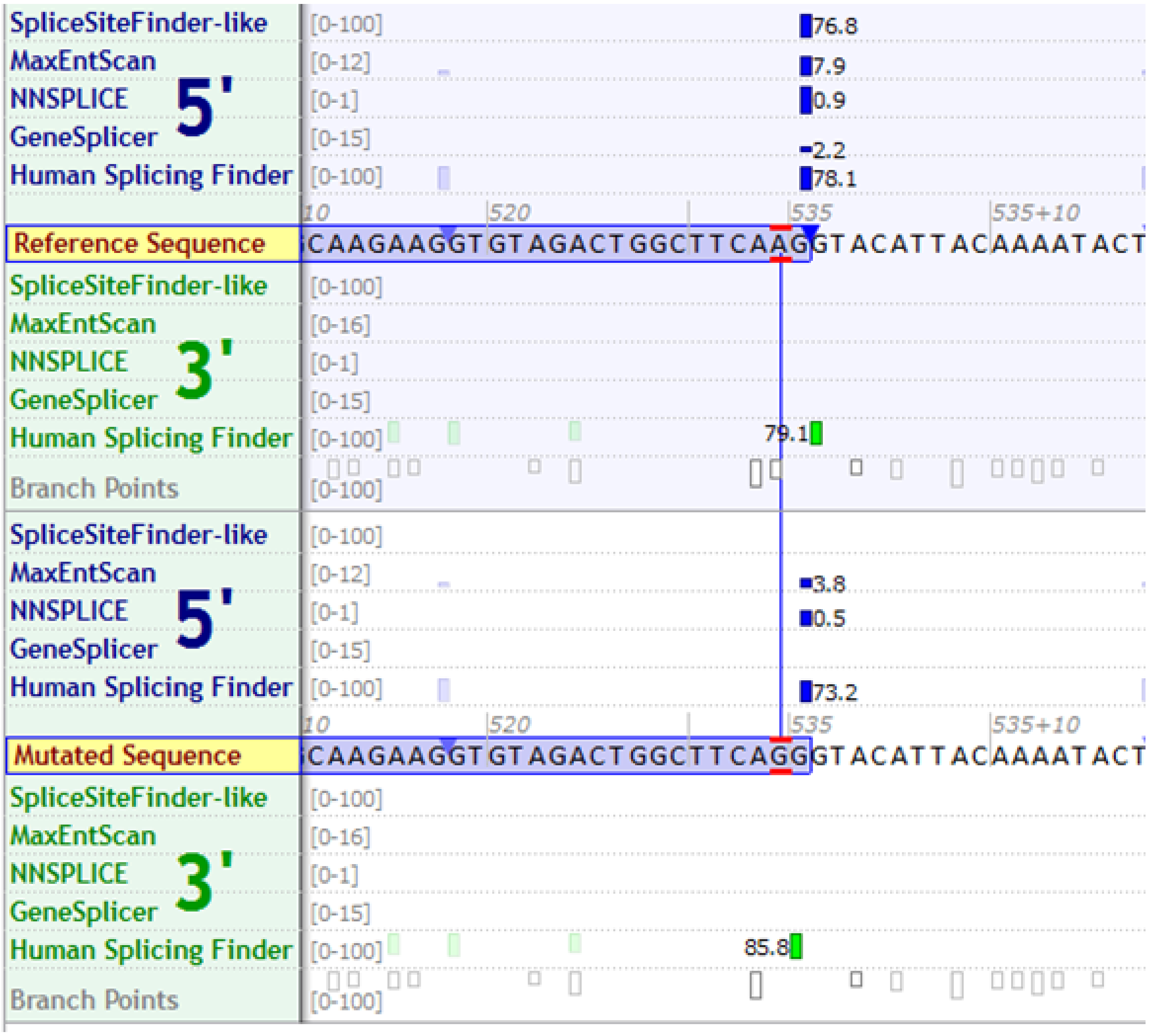

Supplementary Fig. S2: *In silico* analysis of novel synonymous mutation in *ARL6* (c.534A>G; p.=) identified causative factor in family F01. The changes in scores between upper panel (reference sequence) and lower panel (mutated sequence) indicate an effect on splicing of exon 8. Source: Alamut visual Version 2.7.1 from Interactive Biosoftware (http://www.interactive-biosoftware.com).

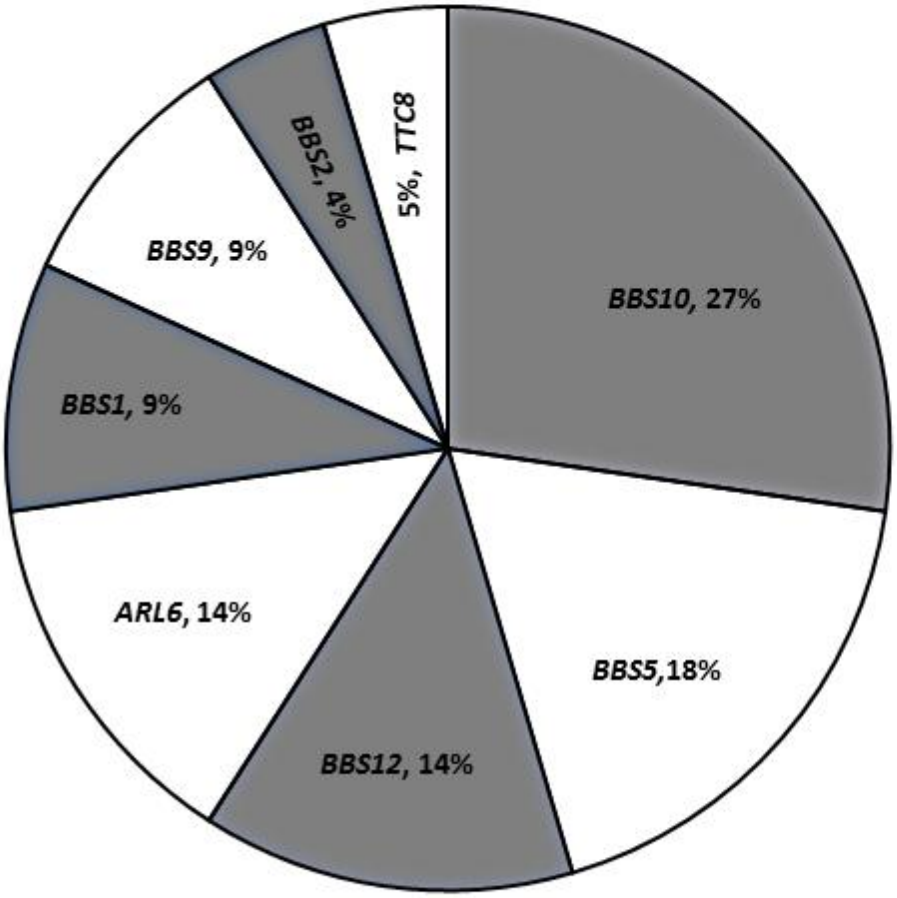

Supplementary Fig. S3: Genetic frequency of eight BBS-associated genes from 22 Pakistani BBS families. Eighteen families were previously reported while 4 BBS families are part of this study, where most of the Pakistani families carry mutations in *BBS10*.
